# Influence of nursing staff attitudes and characteristics on the use of coercive measures in acute mental health services—A systematic review

**DOI:** 10.1111/jpm.12586

**Published:** 2020-01-14

**Authors:** Paul Doedens, Jentien Vermeulen, Lindy‐Lou Boyette, Corine Latour, Lieuwe de Haan

**Affiliations:** ^1^ Department of Psychiatry Amsterdam University Medical Centers, location Academic Medical Center Amsterdam Netherlands; ^2^ ACHIEVE Centre of Applied Research Faculty of Health Amsterdam University of Applied Sciences Amsterdam Netherlands; ^3^ Department of Clinical Psychology University of Amsterdam Amsterdam Netherlands

**Keywords:** coercion, nurse role, safety and security, seclusion and restraint, systematic literature review

## Abstract

**What is known on the subject?:**

- Aggressive behaviour is a major problem in clinical practice of mental health care and can result in the use of coercive measures.
- Coercive measures are dangerous for psychiatric patients and international mental healthcare works on the elimination of these interventions.
- There is no previous review that summarizes the attitude of nursing staff towards coercive measures and the influence of nursing staff characteristics on attitude towards and the use of coercive measures.

**What the paper adds to existing knowledge?:**

- The attitude of nurses shifted from a therapeutic paradigm (coercive measures have positive effects on patients) to a safety paradigm (coercive measures are undesirable, but necessary for the wards’ safety).
- Nurses express the need for less coercive interventions to prevent seclusion and restraint, but their perception of intrusiveness is influenced by how often they use specific coercive measures.
- The knowledge from scientific literature on the influence of nursing staff on coercive measures is highly inconclusive, although the feeling of safety of nurses might prove to be promising for further research.

**What are the implications for practice?:**

- There is need for increased attention specifically for the feeling of safety of nurses, to better equip nurses for their difficult work on acute mental health wards.

**Abstract:**

**Introduction**

The use of coercive measures generally has negative effects on patients. To help prevent its use, professionals need insight into what nurses believe about coercion and which staff determinants may influence its application. There is need for an integrated review on both attitude and influence of nurses on the use of coercion.

**Aim**

To summarize literature concerning attitude of nurses towards coercive measures and the influence of staff characteristics on the use of coercive measures.

**Method**

Systematic review.

**Results**

The attitude of nurses changed during the last two decades from a therapeutic to a safety paradigm. Nurses currently view coercive measures as undesirable, but necessary to deal with aggression. Nurses express the need for less intrusive interventions, although familiarity probably influences its perceived intrusiveness. Literature on the relation between staff characteristics and coercive measures is inconclusive.

**Discussion**

Nurses perceive coercive measures as unwanted but still necessary to maintain safety on psychiatric wards. Focussing on the determinants of perception of safety might be a promising direction for future research.

**Implications for practice**

Mental health care could improve the focus on the constructs of perceived safety and familiarity with alternative interventions to protect patients from unnecessary use of coercive interventions.

## 1 INTRODUCTION

Aggressive behaviour is a broad behavioural construct that includes the concept of violence and causes safety issues in mental health care (Gaynes et al., 2017; Liu, 2004). The definition of violence is an act including physical force such as slapping, punching, kicking and biting; use of an object as a weapon; aggressive behaviour such as spitting, scratching and pinching; or a verbal threat involving no physical contact (Nolan, Soares, Dallender, Thomsen, & Arnetz, 2001). The prevalence of physical violence of patients during psychiatric admission differs in Western countries between 7.5% and 15% (Cornaggia, Beghi, Pavone, & Barale, 2011). To protect patients and staff on psychiatric wards from harm caused by violence, professionals use coercive measures, such as seclusion, restraint and compulsory medication (Cowman, Bjorkdahl, Clarke, Gethin, & Maguire, 2017). In Europe, some countries use seclusion as a “preferred” intervention of last resort in case of dangerous situations, while others resort to physical or mechanical restraint (Bak & Aggernaes, 2012). Coercive measures have no therapeutic value and can result in post‐traumatic stress and severe physical injuries for patients (Frueh et al., 2005; Nath & Marcus, 2006; Rakhmatullina, Taub, & Jacob, 2013; Sailas & Fenton, 2000; Steinert, Birk, Flammer, & Bergk, 2013). Consequently, prevention of coercive measures has become a priority of care professionals, researchers and policymakers in mental health services. The international mental health community developed several quality improvement projects in the last few years to diminish its use (Bierbooms, Lorenz‐Artz, Pols, & Bongers, 2017; Bowers, 2014; Duxbury et al., 2019; Lombardo et al., 2018).

To help prevent the use of coercive measures, it is important to know about variables that are predictive for its use. In their systematic review on patient and staff characteristics associated with higher use of restraint, Beghi, Peroni, Gabola, Rossetti, and Cornaggia (2013) reported that male gender, young age, foreign ethnicity, involuntary admission, diagnosis of schizophrenia and presence of male staff were variables associated with more use of restraint. Laiho et al. (2013) described the influence of the previous experience of nurses with coercion on the decision to use coercive measures. The attitude of nurses towards coercive measures is also important. In their systematic review on nurses’ attitudes towards coercion, Happell and Harrow (2010) found a contradiction between practice of seclusion and attitudes and beliefs of nurses about its efficacy and appropriateness. Nurses acknowledged that seclusion had a negative impact on service users, but inpatient violence justified its use (Happell & Harrow, 2010). This is in line with other review studies, such as Riahi, Thomson, and Duxbury (2016) and Laukkanen, Vehviläinen‐Julkunen, Louheranta, and Kuosmanen (2019) who concluded that coercive measures are still seen as necessary measure of “last resort,” although the attitude of nurses is turning increasingly negative. Furthermore, Riahi et al. (2016) suggest that staff composition and nurses’ perception are important themes in the decision‐making process towards the use of coercive measures. Happell and Harrow (2010) suggest that future research needs to consider staff characteristics together with attitude towards seclusion. Currently, a systematic review that evaluates both the attitude of nurses and the influence of nursing staff characteristics related to coercive measures is lacking.

## 2 AIMS

The aim of this paper is to summarize scientific literature concerning the attitude of nurses towards coercive measures and the influence of nursing staff characteristics on both the use of and the attitude towards coercive measures in acute mental health services. Our research questions are as follows: (a) What are the attitudes of psychiatric nurses towards use of coercive measures? and (b) Which individual or team nursing staff characteristics are associated with the use of coercive measures and with the attitude of nurses towards coercive measures in acute mental health services?

## 3 METHODS

### 3.1 Design

We performed a systematic review and used the PRISMA statement to guide our reporting (Moher, Liberati, Tetzlaff, & Altman, 2009). We defined attitude towards coercive measures according to Bowers et al. (2007) p.358 as “the pattern of beliefs, judgements and feelings about coercive measures.” We divided nursing staff characteristics into individual characteristics (e.g., gender, age, personality traits), professional characteristics (e.g., education, work experience) and organizational characteristics (e.g., staff–patient ratio).

### 3.2 Search

We performed electronic searches in MEDLINE (via OvidSP, 1946—14 March 2019), Embase (via OvidSP, 1947—14 March 2019), PsycINFO (via OvidSP 1880—14 March 2019) and CINAHL Plus (1937—14 March 2019). We describe the full search strategy in Data S1. A clinical librarian assisted with our search. We used no restrictions on language or publication date. We searched reference lists of previous reviews and included studies to find additional publications. We also searched trial registers for registered cohort studies.

### 3.3 Study selection

We performed the first selection based on title and abstract. We subsequently retrieved the full text of the included studies for the final assessment of eligibility. Two reviewers (PD and JV) performed the selection independently and settled disagreements through discussion. In case of disagreement, the reviewers consulted a third reviewer (CL).

We selected studies based on inclusion and exclusion criteria. Inclusion criteria concerning study design were cohort studies, case–control studies, case series, cross‐sectional studies, surveys and qualitative studies on the attitude of nursing staff towards coercive measures and/or the influence of nursing staff characteristics on the use of one or more coercive measures (seclusion, mechanical restraint, physical restraint and compulsory medication). We included studies performed in acute mental health inpatient services or psychiatric facilities in general or academic hospitals that cared for psychiatric patients with primary diagnosis of axis I or II of the DSM‐IV‐TR (American Psychiatric Association, 2000), except addiction disorders and learning disabilities or their equivalent in the DSM‐5 (American Psychiatric Association, 2013). Studies that included also other professionals (such as physicians) and other settings (such as forensic wards) were included if the majority (>50%) of the staff members or settings met our inclusion criteria. We excluded studies performed solely in forensic, child, adolescent and geriatric psychiatry, in general hospital wards, emergency departments, nursing homes or with an outpatient patient population. We excluded studies that addressed aggressive behaviour as outcome measure. We also excluded reviews, case reports, theses, conference abstracts and non‐empirical publications, such as editorials.

### 3.4 Assessment of the risk of bias

We used the Quality in Prognostic Studies (QUIPS) tool (Hayden, van der Windt, Cartwright, Cote, & Bombardier, 2013) for cohort studies, the Newcastle–Ottawa Scale (NOS) (Wells et al., 2000) for case–control studies and the Consolidated criteria for reporting qualitative research (COREQ) (Tong, Sainsbury, & Craig, 2007) for qualitative research.

### 3.5 Data extraction and analysis

Two independent reviewers (PD & JV) performed the data extraction with a standardized form. Studies that described the attitude of nurses were mostly qualitative or survey studies, and the results were not suitable for statistical pooling. We carefully read the studies and extracted important themes from these studies independently. Thereafter, we discussed the interpretation of the qualitative findings. Subsequently, we extracted descriptive themes from the analysis of the qualitative studies based on consensus between the reviewers and combined these with the results from the surveys. We observed that literature on nursing staff characteristics had high levels of heterogeneity, which made it unlikely that performing a meta‐analysis would be appropriate. We summarized the most important results of the included studies. We extracted data on the research question, design, sample size, population, setting and outcome measures from the included studies.

## 4 RESULTS

### 4.1 Search results and quality assessment

**Figure 1:**
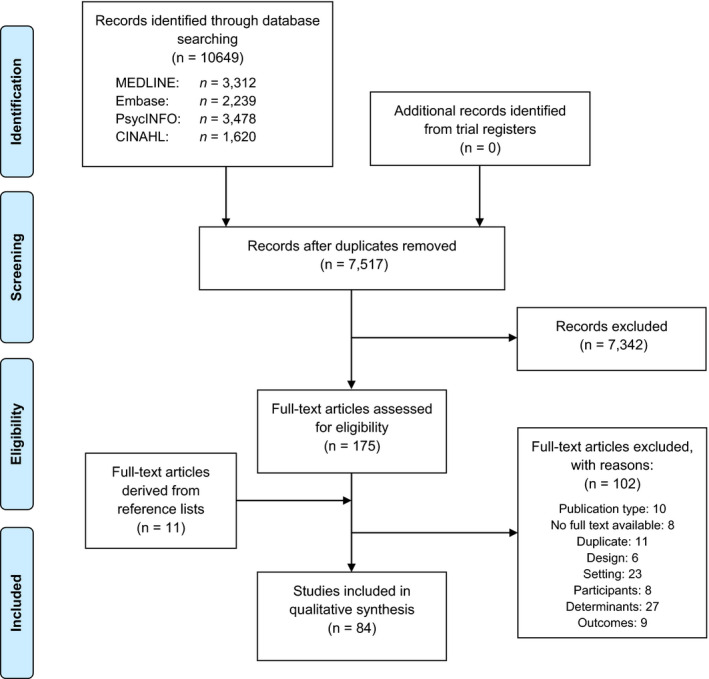
PRISMA flow diagram

The initial search resulted in 7,517 references. After the selection process, we included 84 publications (Figure 1). Among these were papers written in English (78), Dutch (2), German (2) and French (2). Sixty of these papers were reported on the attitudes of nurses and 31 papers reported on the influence of nursing staff characteristics. The data of a large cross‐sectional study from the United Kingdom, named City‐128, accounted for seven publications (Bowers, 2009; Bowers & Crowder, 2012; Bowers, Nijman, Simpson, & Jones, 2011; Bowers, Stewart, Papadopoulos, & Iennaco, 2013; Bowers et al., 2010; Bowers, Van Der Merwe, Paterson, & Stewart, 2012; Whittington, Bowers, Nolan, Simpson, & Neil, 2009). A cross‐sectional study from Norway accounted for two publications (Husum, Bjorngaard, Finset, & Ruud, 2010, 2011), and a survey from Australia accounted for two publications (Happell & Koehn, 2010, 2011). These papers were not duplicates, but described different analyses based on a single, large data set. Therefore, we included 76 unique studies in our review, of which four were prospective cohort studies, five were retrospective cohort studies, four were case–control studies, one was a mixed‐method study, nine were cross‐sectional studies, 31 were surveys and 22 were qualitative studies. These studies originated from 25 different countries. We provide an overview of the included studies in Data S2.

The quantitative studies showed large clinical and methodological heterogeneity. Most of the studies were cross‐sectional studies or surveys based on questionnaires. Several of these studies used self‐developed questionnaires of which the psychometric properties were unknown. Others used validated questionnaires, mostly the Attitudes Toward Seclusion Survey (Heyman, 1987) and the Attitudes to Containment Measures Questionnaire (Bowers, Alexander, Simpson, Ryan, & Carr‐Walker, 2004). Sample size varied from very small (e.g., questionnaire administered with *n* = 13 nurses (Tooke & Brown, 1992)) to very large (e.g., cross‐sectional study with *n* = 11,128 admissions over 136 psychiatric wards (Bowers, 2009)). The available cohort studies and case–control studies often had methodological limitations, such as small sample sizes, retrospective design, limited information on the sampling procedure and data collection on a single ward or hospital. Most of the studies from the eighties and early nineties presented no comprehensive description of the method, statistics and results. The majority of the qualitative studies were of moderate quality. The comprehensiveness of reporting of qualitative studies showed substantial improvement in the last decades, especially in methodological rigour.

### 4.2 Attitudes towards coercive measures

In our study of the included literature on the attitudes of nurses towards coercive measures, we observed two major themes: (a) the discrepancy between treatment paradigm and safety paradigm; and (b) the need for less intrusive alternative interventions.

#### 4.2.1 *Treatment paradigm *versus* safety paradigm*

We observed a paradigm shift in the attitude towards coercive measures from a treatment paradigm to a safety paradigm. The belief that patients experience therapeutic benefits from the use of coercive measures characterizes the treatment paradigm. Distinctive for the safety paradigm is the belief that the patient undergoing coercive measures experiences negative consequences, but coercive measures are necessary to maintain safety for patients and staff members.

Tooke and Brown (1992) were the first to report attitudes of nurses from the therapeutic paradigm and found that nurses believed seclusion was a calming, therapeutic experience. Coercive measures were seen as effective interventions to protect patients’ dignity (Palazzolo, Favre, Halim, & Bougerol, 2000). Nurses considered seclusion of violent patients potentially beneficial for other patients and believed seclusion had a calming effect on the secluded patients (Meehan, Bergen, & Fjeldsoe, 2004; Roberts, Crompton, Milligan, & Groves, 2009; Wynaden et al., 2001).

After 2010, reports that supported the therapeutic paradigm became scarce, although it seems clear that a minority of nurses still view coercive measures as calming for specific types of patients (Fereidooni Moghadam, Fallahi Khoshknab, & Pazargadi, 2014; Korkeila, Koivisto, Paavilainen, & Kylma, 2016; Larsen & Terkelsen, 2014). Differences of opinion and moral dilemmas among nurses were reported (Goulet & Larue, 2017; Korkeila et al., 2016; Larsen & Terkelsen, 2014).

An early example of the safety paradigm was DiFabio (1981), who reported that although nurses had numerous emotional and negative experiences with restraint, its use was necessary to control patients’ behaviour in case of dangerous situations. Lendemeijer (1997) stated that the safety of psychiatric wards prevailed over the individual patient's interest and therefore seclusion was required. The necessity of using seclusion and other coercive measures in case of aggressive behaviour, despite doubts on the therapeutic effect, was also reported by several other authors during the nineties (De Cangas, 1993; Holzworth & Wills, 1999; Muir‐Cochrane, 1996; Olofsson, Gilje, Jacobsson, & Norberg, 1998). In the following decade, nurses reported feelings such as disapproval, failure, guilt and regret after using coercive measures (Bonner, Lowe, Rawcliffe, & Wellman, 2002; Gelkopf et al., 2009; Haglund, Von Knorring, & Von Essen, 2003; Marangos‐Frost & Wells, 2000; Roberts et al., 2009; Wynaden et al., 2001; Wynn, 2003). Bigwood and Crowe (2008) stated that physical restraint was undesirable but unavoidable: “it's part of the job, but spoils the job.” Lemonidou et al. (2002) found that nurses had “positive” attitudes towards seclusion, but mainly because they viewed seclusion as necessary, not desirable. Nurses viewed seclusion as effective for controlling “difficult situations,” but also expressed their concerns about negative consequences for patients (Lee et al., 2003). From 2010, the paradigm shifted more and more towards coercive measures being a “necessary evil,” rather than a therapeutic tool (Wilson, Rouse, Rae, & Kar Ray, 2017). Numerous studies reported that nurses considered coercive measures unwanted and harmful, but necessary to regain safety in the case of aggressive behaviour (Fereidooni Moghadam et al., 2014; Gerace & Muir‐Cochrane, 2019; Guivarch & Cano, 2013; Happell et al., 2012; Happell & Koehn, 2010, 2011; Khalil, Al Ghamdi, & Al Malki, 2017; Khudhur, 2013; Larsen & Terkelsen, 2014; Mahmoud, 2017; Mann‐Poll, Smit, Koekkoek, & Hutschemaekers, 2015; Muir‐Cochrane, O'Kane, & Oster, 2018; Okanli, Yilmaz, & Kavak, 2016; Perkins, Prosser, Riley, & Whittington, 2012; Van der Nagel, Tuts, Hoekstra, & Noorthoorn, 2009; Vedana et al., 2017; Wilson et al., 2017).

In sum, the necessity of coercive measures for dealing with danger due to aggressive behaviour of patients seems a key element of the current attitude of nurses.

#### 4.2.2 Need for less intrusive alternative interventions

Our second theme observed in the studies about nursing staff's attitude was the need for alternative interventions to maintain the safety of patients and staff on psychiatric wards.

The shift from the treatment to the safety paradigm is a key factor in the need for alternatives. Despite the negative consequences and feelings, nurses feared elimination of coercive measures as a tool for dealing with aggressive behaviour and expressed concerns that society will blame them in the future for using coercion and for the negative consequences of not using coercion (Muir‐Cochrane et al., 2018). Because of the perceived necessity of using coercive measures, alternative interventions are vital to align with the ambition to diminish their use from mental health care. Specifically, nurses seem to perceive the severity of coercive interventions as something that needs attention.

Nurses expressed the desire for more “gentle” interventions to manage patients’ behaviour (Olofsson et al., 1998). To make coercion more humane, nurses believed that the practice of coercive measures needed to improve, for example by making the seclusion room more comfortable (Happell et al., 2012; Happell & Koehn, 2010; Jacob, Holmes, Rioux, Corneau, & MacPhee, 2017). Several studies recognized that nurses view seclusion and restraint only as appropriate as intervention of “last resort,” when other interventions have failed (Gelkopf et al., 2009; Goulet & Larue, 2017; Guivarch & Cano, 2013; Happell & Koehn, 2011; Jacob et al., 2017; Khudhur, 2013; Marangos‐Frost & Wells, 2000; McCain & Kornegay, 2005; Palazzolo et al., 2000; Terpstra, Terpstra, Pettee, & Hunter, 2001; Wilson et al., 2017; Wynaden et al., 2002; Wynn, Kvalvik, & Hynnekleiv, 2011). However, the concept of “last resort” is unclear and some staff members viewed the point that an intervention is “of last resort” earlier than others did (Happell et al., 2012; Wilson et al., 2017). Seclusion and restraint have major impact on the patient, and nurses were generally concerned about their well‐being when applying these interventions (Lee et al., 2003; Wynn, 2003).

Although seclusion and restraint are both seen as highly intrusive, several authors reported that nurses viewed seclusion and forced medication as less intrusive and, thus, favourable compared to mechanical restraint (Gerace & Muir‐Cochrane, 2019; Guivarch & Cano, 2013; Jacob et al., 2017; Larsen & Terkelsen, 2014). Other authors stated that nurses preferred the use of the least intrusive intervention when considering the use of coercive measures, such as pro re nata (PRN or as needed) medication (Bennett, Ramakrishna, & Maganty, 2011; Gelkopf et al., 2009; Khalil et al., 2017; Meehan et al., 2004; Reisch et al., 2018; Terpstra et al., 2001) and close observation or individual counselling (Bennett et al., 2011; Holzworth & Wills, 1999; Muir‐Cochrane, 1996; Palazzolo et al., 2000).

The frequency of use also influenced the perceived intrusiveness of coercive interventions. Whittington et al. (2009) used the sample of City‐128 to assess the view of nurses towards eleven forms of coercive measures (locked‐door seclusion, open‐area seclusion, mechanical restraint, physical restraint, net bed, transfer to a psychiatric intensive care unit (PICU), time out, constant observation, intermittent observation, consensual PRN medication and compulsory intramuscular medication) on six domains (effectiveness, acceptability, respectfulness, safety for service users, safety for staff and willingness to use the measure). The three interventions with least approval of staff were net beds, mechanical restraint and open‐area seclusion. These interventions were not (net beds and mechanical restraint) or seldom (open‐area seclusion) used in mental health services in the UK. The three methods with most approval (transfer to the PICU, PRN medication and observation) were considered common practice (Whittington et al., 2009). Therefore, nurses showed low rates of approval for coercive measures they seldom or never use and report more favourably on familiar practices. Özcan, Bilgin, Akin, and Badirgali Boyacioglu (2015) supported this finding. They found a correlation between the frequency of use of coercive measures and positive attitudes towards the coercive measure. Van Doeselaar, Sleegers, and Hutschemaekers (2008) found that nurses who are more actively involved in use of seclusion had less ethical concerns for seclusion than non‐involved professionals, such as psychologists and therapists. Gerace and Muir‐Cochrane (2019) suggested that nurses were supportive towards the elimination of mechanical restraint use because they are less frequent than other coercive measures. Dahan et al. (2018) reported that participants who were present during mechanical restraint practices had more positive attitudes than participants who were never present. Pettit et al. (2017) found that availability of a coercive measure was associated with approval of the use of the coercive measure. For example, access to a seclusion room was associated with greater acceptability of seclusion as a method of containment (Pettit et al., 2017).

In sum, nurses consider seclusion and restraint generally as most intrusive interventions and express the need for less intrusive alternatives to diminish their use. The attitude of nurses towards specific coercive measures seems more positive for interventions used more frequently in practice.

### 4.3 Influence of nursing staff characteristics

Next, we summarize the results of the quantitative studies on the influence of nursing staff characteristics (individual, professional and organisational) on the use of and attitude towards coercive measures.

#### 4.3.1 Individual characteristics

Gender of the nurse is the most reported nursing staff characteristic associated with use of and attitude towards coercive measures, although findings are inconsistent. Several studies reported that the presence of male nurses was associated with more use of coercive measures, such as seclusion (Bowers et al., 2010; De Cangas, 1993; Morrison & Lehane, 1995) or restraint (Kodal, Kjaer, & Larsen, 2018). Male nurses also showed more positive attitudes than female nurses towards coercive measures (Bregar, Skela‐Savic, & Kores Plesnicar, 2018; Husum, Bjorngaard, Finset, & Ruud, 2011; Khalil et al., 2017; Lind, Kaltiala‐Heino, Suominen, Leino‐Kilpi, & Valimaki, 2004; Mohammed, 2015; Whittington et al., 2009) Male nurses were found to be more supportive of coercive measures after “bad behaviour” or damaging property (Gelkopf et al., 2009; Happell & Koehn, 2010). However, other studies reported that the presence of female nurses was associated with more seclusion (Convertino, Pinto, & Fiester, 1980; Janssen, Noorthoorn, Linge, & Lendemeijer, 2007) or restraint (Bornstein, 1985) and that female gender is associated with more positive attitudes towards coercive measures (Gandhi et al., 2018; Hasan & Abulattifah, 2018; Jonker, Goossens, Steenhuis, & Oud, 2008; Wynn, 2003). Bowers et al. (2013) reported that wards with high levels of aggression and low use of coercive measure seemed to have less female staff members. Other studies found no associations in (multivariable) analysis between gender of the nurse and use of coercive measures (Bowers, 2009; De Benedictis et al., 2011; Doedens et al., 2017; O'Malley, Frampton, Wijnveld, & Porter, 2007; Vollema, Hollants, Severs, & Hondius, 2012).

Several authors investigated nurses’ age in relation to use of seclusion, but found no associations (Bowers et al., 2012; De Benedictis et al., 2011; Doedens et al., 2017; Kodal et al., 2018). Some authors reported that young age was associated with more positive attitudes towards seclusion (Happell & Koehn, 2010; Wynn, 2003) or coercive measures in general (Husum et al., 2011), although an opposite effect was found for physical restraint (Wynn, 2003).

The City‐128 study investigated ethnicity of the nurse and found that the proportion of white staff members in a team was associated with more use of coercive measures, compared with African and other ethnicities (Bowers, 2009). De Benedictis et al. (2011) examined the role of religion and non‐native Canadian nurses and found no associations on both accounts. The variables physical stature and BMI were both reported as not associated with seclusion (Doedens et al., 2017).

A creative personality, measured on Gough's Adjective Checklist (Gough, 1960), and high leadership scores, measured on Kolb's Organizational Climate Questionnaire (Kolb, Rubin, & McIntyre, 1971), were found to be associated with less initiation of coercion (Pawlowski & Baranowski, 2017). High scores on transactional leadership, measured as a subscale of the Multifactor Leadership Questionnaire (Bass & Avolio, 1995), were also found to be associated with less use of coercive measures (Bowers, 2009). Staff members with high empathy scores (scored on a scale of one (below‐average empathy) to five (above average empathy)) were less prone to use seclusion and restraint (Yang, Hargreaves, & Bostrom, 2014). Happell and Koehn (2011) reported that approval of seclusion for deviant patient behaviour was associated with high scores of emotional exhaustion (measured with the Maslach Burnout Inventory [MBI] (Maslach & Jackson, 1981)) and low scores of therapeutic optimism (nurses’ optimism related to treatment outcomes for patients, measured with the Elsom Therapeutic Optimism Scale (Elsom & McCauley‐Elsom, 2008)). There was no association between anger of nurses and the incidence of seclusion and restraint (Jalil, Huber, Sixsmith, & Dickens, 2017). Bowers (2009) did not find an association between score on the MBI and the use of coercive measures.

Feelings of safety of nurses were likely to be associated with the use of coercive measures, although definition and measurement is complicated. Moreover, direction of causality is mostly unknown. Higher subjective feeling of safety of nurses was associated with less seclusion (Vollema et al., 2012). These authors measured the feeling of safety at the end of each shift. Therefore, an aggressive incident that led to seclusion during the shift may have caused a lower feeling of safety. The feeling of safety was negatively influenced by physical environment (e.g., lack of safety equipment), organisational factors (e.g., low staff–patient ratio), lack of communication with hospital security, patient characteristics and trust within teams, while aggression management training, work experience and information about patients contributed to the feeling of safety (Goulet & Larue, 2017). Goulet and Larue (2017) also described that being a victim or witness of patient assault made nurses feel less safe and may even induced hypervigilance. Gray and Diers (1992) suggested that a decrease in staff stress and increase in feelings of control by staff was associated with an increase in the use of coercive measures, while referring to the “reverse hypothesis” (patient will not act out when staff members are upset). These authors measured staff stress and coercive measures before and after a major organisational change, making it likely that the organizational change caused confounding. Nurses that were assaulted and injured by patients decided to use restraint later in the course of an aggressive incident than nurses that were never injured by patients (Moylan & Cullinan, 2011). A positive attitude towards patients with personality disorders was associated with less seclusion, but not with other forms of coercion (Bowers, 2009; Bowers et al., 2010, 2012).

#### 4.3.2 Professional characteristics

Several authors investigated the educational level of nurses in relation to the use of coercive measures. The City‐128 study divided staff members into qualified and non‐qualified staff. Wards with more qualified staff were associated with more use of seclusion (Bowers et al., 2010). This seemed also to be the case for mechanical restraint (Bowers et al., 2012). Khalil et al. (2017) also reported that higher level of nursing education is associated with more use of seclusion. However, Miodownik et al. (2019) reported a negative association between the presence of academic registered nurses and the duration of coercive measures. The presence of student nurses on a ward was also associated with more mechanical restraint (Bowers et al., 2012). However, most studies that incorporated educational level of nurses in their model found no association with the use of coercive measures (Bornstein, 1985; De Benedictis et al., 2011; Doedens et al., 2017; Janssen et al., 2007; Kodal et al., 2018).

Several authors reported no association between the work experience of nurses and the frequency of use of coercive measures (De Benedictis et al., 2011; Doedens et al., 2017; Janssen et al., 2007; Khalil et al., 2017; Kodal et al., 2018; O'Malley et al., 2007). Janssen et al. (2007) found an association between more variability in the nursing team of a shift and less frequent use of seclusion. Morrison and Lehane (1995) suggested that more experienced nurses (“charge nurses”) might be associated with less use of seclusion, although they did not perform any statistical testing. Some authors suggested that experienced nurses tended to have less supportive attitudes towards the use of coercive measures (Gelkopf et al., 2009; Happell & Koehn, 2010; Korkeila et al., 2016). However, Gandhi et al. (2018) and Bregar et al. (2018) reported more positive attitudes for restraint of nurses with more work experience. Mann‐Poll et al. (2015) found that experienced nurses rated the use of seclusion equally appropriate and necessary, while less experienced nurses showed more ambivalence in necessity and appropriateness.

There is no evidence for an association between the amount of fulltime nurses in a team (De Benedictis et al., 2011; Doedens et al., 2017), the length of time that nurses are working at the ward (Doedens et al., 2017) or their training in aggression management (De Benedictis et al., 2011; Khalil et al., 2017) and the frequency of use of coercive measures.

#### 4.3.3 Organisational characteristics

Staff–patient ratio has received extensive attention in scientific research in the last 30 years. Several authors reported an association between a lower staff–patient ratio (i.e., less staff members for each patient) and an increase in the use of coercive measures (Convertino et al., 1980; Donat, 2002; Morrison & Lehane, 1995; O'Malley et al., 2007). On the contrary, Bowers and Crowder (2012) found that more qualified staff members in the shifts and in the shifts prior to the incident were associated with more frequent use of coercive measures. Fukasawa, Miyake, Suzuki, Fukuda, and Yamanouchi (2018) found a small association between higher staff–patient ratio and an increase in the use of seclusion and restraint. Other authors found no association for staff–patient ratio and the use of coercive measures (Bowers, 2009; Bowers et al., 2010, 2012; Husum, Bjorngaard, Finset, & Ruud, 2010; Janssen et al., 2007; Khalil et al., 2017; Kodal et al., 2018; Sercan & Bilici, 2009; Vollema et al., 2012; Yang et al., 2014) or reported no outcome measurement despite the fact that they mentioned measuring this variable in the method section (Betemps, Somoza, & Buncher, 1993). Klimitz, Uhlemann, and Fahndrich (1998) reported no association between the use of restraint and shortage of nursing staff. The staff–patient ratio varied in most studies of different shifts (day, evening and night). According to Klimitz et al. (1998) and Morrison and Lehane (1995), the night shift has the least use of coercive measures compared to the other shifts. However, other studies found that the night shift has most use of coercive measures compared with other shifts (Convertino et al., 1980; O'Malley et al., 2007). Several authors claim that most coercive measure occurred during the evening shift (Klimitz et al., 1998; Kodal et al., 2018; Reitan, Helvik, & Iversen, 2018). Yang et al. (2014) report substantial higher odds of seclusion in evening, weekend or holiday shifts compared to weekday shifts, but no difference between night shifts and weekday shifts. O'Malley et al. (2007) found no difference of the use of seclusion and the day of the week. Reitan et al. (2018) reported most frequent use of pharmacological restraints during summer and most use of mechanical restraint during spring.

De Benedictis et al. (2011) found that seclusion and restraint occurred more at psychiatric emergency departments or intensive care units than at regular psychiatric wards, but less frequent in non‐teaching hospitals compared to teaching hospitals. The availability of (and compliance to) aggression management protocols was not associated with the use of seclusion and restraint (De Benedictis et al., 2011). Changing a 20‐bed unit into two ten‐bed units (while holding the staff–patient ratio stable) seemed to decrease the use of seclusion, suggesting that deviant patient behaviour can be managed better at small wards (O'Malley et al., 2007).

A higher score on the subscale programme clarity of the Ward Atmosphere Scale (Moos, 1974), indicating an effective structure on the ward, was associated with less use of coercive measures (Bowers, 2009; Bowers et al., 2012). Bowers et al. (2011) divided a sample of 134 wards into two clusters based on their scores on leadership, teamwork, ward atmosphere, burnout levels and attitude towards patients with a personality disorder. The cluster with the highest (positive) scores (*n* = 78) showed less use of coercive measures compared to the clusters with lowest scores (*n* = 56).

Other authors found no association between ward atmosphere and frequency of use of coercive measures (De Cangas, 1993; Klimitz et al., 1998). Bowers (2009) found no association between team climate and the use of coercive measures, contrary to De Benedictis et al. (2011) who reported an association of the subscale anger and aggression of the Group Environment Scale (Moos, Shelton, & Petty, 1973) and the use of seclusion and restraint.

## 5 DISCUSSION

This systematic review aimed to summarize the scientific literature on attitudes of nurses towards coercive measures and on the association between nursing staff characteristics and the use of coercive measures and the attitude of nurses towards coercive measures in acute mental health services.

With respect to the first aim, we observed two major themes in the attitude of nurses towards use of coercive measures. Firstly, the abandonment of a treatment paradigm towards a safety paradigm. In the therapeutic paradigm nurses considered coercive measures as harsh, but helpful, for example calming the agitated patient and protecting patients’ dignity (Lendemeijer, 1997; Palazzolo et al., 2000; Tooke & Brown, 1992). The support for the therapeutic paradigm in the attitude of nurses decreased substantially in the last decades and shifted to the safety paradigm. In the safety paradigm, staff members consider coercive measures a measure of last resort and there is a preference for the least intrusive intervention. This resulted in a strong conflict for nurses, because they consider coercive measures as necessary, but its application inflicts strong negative feelings. This finding is in line with other reviews on attitude towards coercion (Laukkanen et al., 2019; Riahi et al., 2016). Most current research on the attitude of nurses towards coercion shows that nurses viewed coercive measures mainly from the safety paradigm, although the therapeutic paradigm in the attitude of nurses has not disappeared completely (Van Der Merwe, Muir‐Cochrane, Jones, Tziggili, & Bowers, 2013).

The second theme was an expressed need for less intrusive alternative interventions. The increase in the need for less intrusive interventions is consistent with the attitude change to the therapeutic paradigm. Coercive measures are seen as (in the words of Bigwood and Crowe (2008)): “undesirable, but unavoidable”. However, the perspective on what is a “less intrusive alternative intervention” shows to be dependent on several contextual factors. We found that some nurses that used mechanical restraint as intervention of last resort tended to consider seclusion as a less intrusive alternative intervention (Gerace & Muir‐Cochrane, 2019; Guivarch & Cano, 2013; Jacob et al., 2017; Larsen & Terkelsen, 2014), while nurses from other studies consider seclusion as highly intrusive and undesirable intervention (De Cangas, 1993; Lemonidou et al., 2002; Roberts et al., 2009; Wynaden et al., 2001). The impact of seclusion on patients in confirmed by Askew, Fisher, and Beazley (2019), who conclude that patients feel vulnerable, neglected and abused when experiencing seclusion. Nevertheless, both restraint and seclusion are the “ultimum remedium” in case of acute dangerous situations on psychiatric wards and most nurses wish to use alternative interventions with less impact on the patient (Bennett et al., 2011; Holzworth & Wills, 1999; Muir‐Cochrane, 1996; Olofsson et al., 1998; Palazzolo et al., 2000). The everyday experience of the nurse with coercive measures in clinical practice seems to have major influence on the perception of intrusiveness and therefore on the appropriateness of an intervention as alternative. A hypothetical explanation of this finding is that the positive attitude makes nurses choose for that specific coercive measure when necessary. However, there are major differences between countries in the use of coercive measures (Bak & Aggernaes, 2012), which makes it unlikely that nurses based their attitude on these differences instead of on history and culture. Another hypothetical explanation is given by Van Doeselaar et al. (2008), suggesting that the frequency of use of a specific intervention can blind the nurses for possible negative consequences and thereby the perceived “intrusiveness” of an intervention drops. This could explain the association between a positive attitude and the frequency of use of a specific coercive measure (Özcan et al., 2015; Whittington et al., 2009). This theory is in line with Laiho et al. (2013), who stated that the threshold to use coercion gets lower when it was accepted as measure to control behaviour. However, the inconsistent findings on the influence of experience of the nurse on seclusion might indicate that acceptance of coercive measures is also influenced by knowledge of and confidence in using alternative interventions. We recommend further exploration of this issue in future research to reveal a possible blind spot of nurses in their attitude towards coercion and coercive measures.

Our second aim was the influence of nursing staff factors on the use of coercive measures and on the attitude of nurses towards coercive measures. The results in literature were remarkably inconclusive. For example, we found twelve studies that investigated the association of gender of the nurse and the use of coercion. Five of them concluded that male nurses were more prone to use coercion (Bowers et al., 2010; De Cangas, 1993; Khalil et al., 2017; Kodal et al., 2018; Morrison & Lehane, 1995), three of them concluded that female nurses were more prone to use coercion (Bornstein, 1985; Convertino et al., 1980; Janssen et al., 2007) and four of them found no effect in multivariable analysis (Bowers et al., 2011; De Benedictis et al., 2011; Doedens et al., 2017; Vollema et al., 2012). Findings on the influence of the attitude towards coercive measures showed similar pattern; male gender was associated with more positive attitudes by six studies (Bregar et al., 2018; Husum et al., 2011; Khalil et al., 2017; Lind et al., 2004; Mohammed, 2015; Whittington et al., 2009) and also associated by four studies with more negative attitudes (Gandhi et al., 2018; Hasan & Abulattifah, 2018; Jonker et al., 2008; Wynn, 2003). Beghi et al. (2013) concluded in their review that male staff were associated with more restraint; our findings show that this conclusion might have been too firm. We found no conclusive evidence for an association of age, religion or the physique of the nurse and the use of coercion (Bowers, 2009; De Benedictis et al., 2011; Doedens et al., 2017; Kodal et al., 2018). Some authors reported an association between personality factors and use and attitude of coercive measures, but the current studies are too small and inconsistent in methodology to draw conclusions. Also, professional characteristics such as work experience, proportion full time workers, time working at the ward or level of training in aggression management showed no clear association with the prevalence of coercion (De Benedictis et al., 2011; Doedens et al., 2017; Janssen et al., 2007; Khalil et al., 2017; Kodal et al., 2018; O'Malley et al., 2007). Some studies show that experienced nurses had less positive attitudes on the use of coercive measures, but these results also are equivocal (Bregar et al., 2018; Gandhi et al., 2018; Gelkopf et al., 2009; Happell & Koehn, 2010; Korkeila et al., 2016; Mann‐Poll et al., 2015). However counterintuitive, several authors suggested that better‐qualified nurses were associated with more use of coercive measures (Bowers et al., 2010, 2012; Khalil et al., 2017). A possible explanation is that wards with more qualified nurses serve a more complex patient population. Again, most authors report no association of nurses’ educational level and the use of coercion (Bornstein, 1985; De Benedictis et al., 2011; Doedens et al., 2017; Janssen et al., 2007; Kodal et al., 2018). Some authors reported that higher staff–patient ratios were associated with less coercion (Convertino et al., 1980; Donat, 2002; Morrison & Lehane, 1995; O'Malley et al., 2007), but most studies reported no association between these factors (Bowers, 2009; Bowers et al., 2010, 2012; Husum et al., 2010; Janssen et al., 2007; Khalil et al., 2017; Sercan & Bilici, 2009; Vollema et al., 2012; Yang et al., 2014).

When combining the findings of the perceived necessity of coercive measures for safety reasons and the inconsistency in the influence of nursing staff characteristics, we want to stipulate the possible importance of the feeling of safety of nurses. Despite the troubles of measuring this trait, some authors suggest that the feeling of safety of nurses may be associated with less use of coercive measures (Goulet & Larue, 2017; Vollema et al., 2012). This is in line with the findings of the nurses’ attitude towards coercion. Nurses that feel unsafe may very well view a coercive measure as necessary to restore safety, while nurses that feel safe may settle for alternative (less coercive) interventions. This is in line with the findings of Cusack, McAndrew, Cusack, and Warne (2016) that staffs’ fear motivates for the use of coercion. Happell et al. (2012) and Wilson et al. (2017) reported nurses that were concerned that some nurses considered the necessity of a “last resort intervention” earlier than others. Feelings of safety or danger are not objective constructs, so interpersonal differences in perception and perspective highly affect the treatment of patients when potential dangerous situations occur. The attention of professionals, researchers and policymakers on the interpretation of the concepts of safety and danger could be crucial for taking next steps in reducing coercive measures.

This current systematic review is, to the best of our knowledge, the first to explicitly combine a review on the attitude of nurses and the influence of nursing staff characteristics. The strengths are that we performed an extensive literature search in several databases and to several forms of coercive measures, instead of focussing on seclusion and restraint. There are also some limitations. Summarizing qualitative studies inevitably entails de‐contextualisation of qualitative findings, because of the dependency of qualitative research findings on the particular context, time and group of participants (Thomas & Harden, 2008). The heterogeneity and methodological limitations of the studies on nursing staff characteristics in associations with coercive measures made it impossible to perform a meta‐analysis. Another limitation is that the concept of attitude is not well defined and that several authors use other words to describe attitude. In our search, we also evaluated studies on perspectives, experiences and views of nurses to find additional studies on this matter. There were also specific limitations applicable to individual studies. The studies were of moderate to low methodological quality, which hinders the validity of the results of this review. Another limitation is that authors tend to report only significant associations or large effect sizes. Because of that, we cannot rule out the underreporting of some characteristics due to publication bias. We extracted the data from manuscripts as thoroughly as possible to summarize all reported (non‐significant) results in our study.

## 6 CONCLUSION

The attitude of nurses towards coercive measures has changed over the years from a therapeutic paradigm to a safety paradigm. The current attitude towards use of coercive measures is not to treat patients, but to protect patients and staff from violence. Nurses consider coercive measures as necessary interventions and express the need for less intrusive alternatives. Although nurses recognize the negative consequences for patients, the frequent use of a specific coercive measure may decrease the value that nurses give to the negative consequences associated with that measure. The research on the influence of nursing staff characteristics is highly inconclusive. However, the feeling of safety of nurses may be a key concept in the prevention of coercive measures.

## 7 IMPLICATIONS FOR PRACTICE

We propose that mental health care could improve the focus on the constructs of safety and danger to protect patients from unnecessary use of coercive interventions. Lack of attention to the feeling of safety of nurses working at psychiatric wards can threaten further reduction in the use of coercive measures. Using coercive measures has been common practice in mental health care for centuries, as well as the debate on reducing them (Yellowlees, 1872). It is part of our culture and, “culture eats strategy for breakfast” (Muir‐Cochrane, 2018). It is important to invest in the feeling of safety of nurses to help them cope with changing the policy on using coercive measures. Evidence‐based intervention programmes such as Safewards (Bowers, 2014) and Six Core Strategies (LeBel et al., 2014) can help nurses gain confidence in doing their job. To develop specific strategies to improve these feelings could be an interesting topic for researchers in the mental health field. Improvement of patient safety relies on qualified nurses that feel safe and are equipped for the difficult task they are facing when working in acute clinical psychiatry.

## 8 RELEVANCE STATEMENT

The use of coercion is associated with adverse events. Nurses have influence on the decision to use coercive measures. Attitude of nurses towards coercion and nursing staff characteristics influence these decisions. This review summarizes the literature on the influence of attitude of nurses and nursing characteristics on the use of coercive measures. Our findings indicate, based on the attitude towards coercive measures and some evidence on perception of safety, the importance of the feeling of safety of nurses by clinicians, researchers and policymakers. This might be a more relevant road towards better quality of care than focus on nursing characteristics.

## CONFLICT OF INTEREST

The authors declare no conflicts of interest.

## AUTHOR CONTRIBUTIONS

All authors listed meet the authorship criteria according to the latest guidelines of the International Committee of Medical Journal Editors. All authors agree with the manuscript.
